# Supplementary material for: Nutritional outcomes of therapeutic feeding program and its predictors among undernourished adult HIV positive patients at healthcare facilities of West Guji Zone, Southern Ethiopia: A retrospective cohort study
Source: PLoS One. 2024 Jan 23;19(1):e0297436. doi: 10.1371/journal.pone.0297436 (PMC10805293; doi:10.1371/journal.pone.0297436)
Supplement: S1 File — (PDF) [file pone.0297436.s001.pdf]

Data extraction tool (English version)

S.no	Questions	Response options	Remark
	Part I. Socio demographic factors		
101	Age		
102	Sex	1 Male	
		2 Female	
103	Educational status	1 No formal education	
		2 Primary	
		3 Secondary	
		4 Higher education	
104	Employment status	1 Self employed	
		2 Government/NGO employee	
		3 unemployed	
105	Marital status	1 Single	
		2 Married	
		3 Divorced	
		4 Widowed	
106	Residence	1Rural	
		2 Urban	
107	Religion	1 Orthodox	
		2 Protestant	
		3 Muslim	
		4 Other	
Part II Clinical and immunological factors			
201	WHO Clinical stage	1 Stage 1 and 2	
		2 Stage 3 and 4	
202	ARV regimen	1 First line	
		2 Second line	
		3 Third line	
203	ART adherence level	1 Good	
		2 Fair	
		3 Poor	
204	Base line OIs	1 Yes	

		2 No	
205	Cotrimoxazole preventive therapy	1 Yes<br>2 No	
206	INH Status	1 Yes<br>2 No	
207	Other medication	1 Yes<br>2 No	
208	Base line<br>CD4 level	1. < 200 cells/m^3	
		2. 200-350 cells/m^3	
		3. >350 cells/m^3	
209	Hemoglobin level	1 < 10	
		2 10-11.99	
		3 >= 12	
210	Functional status	1.Working	
		2.Ambulatory	
		3. Bed ridden	
211	ART status	1 On HAART<br>2 Pre ART	
213	Months on ART	1. < 6 months	
		2. 6 – 12 months	
		3.12 months and above	
214	Base line nutritional status	1 SAM	
		2 MAM	
215	BMI at admission		
216	BMI at discharge		
217	Months on Nutritional treatment		
218	Nutritional treatment outcome	1 Recovered/ graduated	
		2 Defaulter	
		3 No response	
		4 Died	
