# Supplementary material for: Nutritional outcomes of therapeutic feeding program and its predictors among undernourished adult HIV positive patients at healthcare facilities of West Guji Zone, Southern Ethiopia: A retrospective cohort study
Source: PLoS One. 2024 Jan 23;19(1):e0297436. doi: 10.1371/journal.pone.0297436 (PMC10805293; doi:10.1371/journal.pone.0297436)
Supplement: Attachment — Submitted filename: Review report.docx [file pone.0297436.s002.docx]

**Review report**

Thank you very much for the opportunity to review this manuscript that addresses an important clinical subject in its field. However, I feel that there are number issues that need to address as detailed in the attached document.

Comments

- General comments
  - The paper is well written but the language, grammar and punctuation needs to be improved significantly.
- Background
  - This section is uninformative because it does not review the literature on the main issue of the study. The authors mainly focused on the prevalence of HIV particularly, in the first paragraphs, while the main issue is nutritional recovery among HIV/AIDS patients,
- Highlight on the epidemiological burden of undernutrition among people living with HIV/ AIDS in terms of its prevalence / rate and the relationship between the two in the background section.

Results

- Figure 1 is not self-explanatory. The X & Y axes are not labeled.
- Discussion
  - There are same vague statements in the discussion section. For example, the statement that says “Age was a further socio-demographic variable connected to nutritional treatment recovery”. This statement needs to be rephrased.
  - Researchers should write the implications of the main findings of their study the bridge the gap between policy and research. Better to include the implications of the major findings.
- Conclusion
  - Line 469: The statement that says Nutritional recovery in this study fell short of WHO Sphere requirements. I think you mean **falls short** of …
