# Supplementary material for: Nutritional outcomes of therapeutic feeding program and its predictors among undernourished adult HIV positive patients at healthcare facilities of West Guji Zone, Southern Ethiopia: A retrospective cohort study
Source: PLoS One. 2024 Jan 23;19(1):e0297436. doi: 10.1371/journal.pone.0297436 (PMC10805293; doi:10.1371/journal.pone.0297436)
Supplement: Attachment — Submitted filename: Comments to the author_plos.docx [file pone.0297436.s003.docx]

Thank you for inviting me to review the manuscript entitled “Nutritional outcomes of Therapeutic feeding program and its predictors among undernourished adult HIV positive patients at healthcare facilities of West Guji Zone, Southern Ethiopia: A Retrospective Cohort Study” . It would have important contribution for decision makers by suggesting strategies in improving the management of HIV patients which is a significant cause of morbidity and mortality in Ethiopia. But the manuscript is not fit for publication in its current form.

**General comment:**

1. The manuscript has many editorial problems and needs language editing.

**Abstract:**

1. In the background section of the abstract, the problem under study is not well stated. I suggest rewriting it to show what the problem or gap is and why it needs to be studied.
2. The abstract is lengthy and can be made shorter by minimizing unnecessary details in the methods section.
3. The conclusion does not align with the results. For instance, you have conclude that divorced marital status was a negative predictor but you didn’t mention it in the result section.

**Introduction:**

1. Line 58. From the sentence “The human immunodeficiency virus and acquired immune deficiency syndrome……”, each word should be capitalized and put the abbreviation in bracket
2. From Line 67 to 68, the authors mentioned that HIV/AIDS affects 67% of people worldwide. I think this figure is exaggerated and I doubt on the credibility of your source. Check it again.
3. Abbreviations should be written in full words in their first use.
4. There is a problem of coherence. The authors discuss about the treatments of malnutrition first (line 72 to 97), and then they discuss about the magnitude and effect of malnutrition (line 99 to 107). I suggest to discuss the magnitude and effect of malnutrition among HIV patients first.
5. I believe that the problem under study (undernutrition among HIV) is not well stated. Rather the authors focus on the magnitude of HIV and undernutrition treatment procedure.

**Methods:**

1. The study period for a follow-up study should be the time between the initiations of follow up and end of follow up. The authors mentioned the time when they access the patients chart.
2. The study populations of this study were “All under-nourished HIV-infected adults registered in therapeutic feeding centers of public hospitals in West Guji Zone between January 1, 2018 and December 30, 2022” and the data were collected from February 1-30, 2023. But in order to assess whether the participant is recovered or not it needs a minimum of 3 months after initiation of therapeutic feeding based your operational definition. So, there should be at least a 3 months gab between the last date of subject recruitment and outcome assessment. What is your explanation here?
3. In the inclusion criteria, why do the authors need to know the nutritional treatment outcomes in order to include the participants in the study? There could be selection bias if we included participants after knowing their outcome status.
4. The authors didn’t calculate the sample size using appropriate formula for survival analysis.
5. I think the data collectors were health care providers working in the ART clinic. This could introduce observer bias, so how do you manage it?
6. The authors stated that the dependent variable was “nutritional treatment outcome (recovered or unrecovered)”, means it is a binary outcome. So why you conduct survival analysis? If you want conduct a survival analysis, you need to have a time to event data. Please make it clear.
7. The authors operationalized adherence. But is it adherence to ART or Adherence to food by prescriptions? I think both are important
8. The authors need to define the time to event and censor if they want to stick with survival analysis. They didn’t mentioned when they start and stop following the participants.

**Results:**

1. The minimum and maximum follow up period, median follow up period, and the total person time contribution of the study participants were not mentioned.
2. Make tables up to the standard by reducing lines
3. When I read the result section, I am confused about what the event is. The authors try fit 3 different models considering 3 events, recovery, non-response and default. You have to stick with one of it. Which one is the problem that you are interested in? Then, the introduction and the methods will be also related with the event of interest.

**Discussion:**

1. Start by summarizing your finding, for instance line 404 you just start with comparison
2. The discussion is poorly written which lacks adequate comparison and justifications.
3. From line 464 to 465, the authors mentioned the following as a limitation. *“The absence of a control group for comparison and the possibility of data recruitment bias resulted from the samples being restricted to only people with recorded nutritional results.”* How could this limitations affect the study? What kind of control group you could use?

**Conclusion:**

1. **From line 469 t0 471, the authors conclude that “***The likelihood of nutritional recovery is increased by divorce, WHO clinical stages I and II at admission, working functional status, and age between 18 and 39.”* But the result doesn’t support that divorce increases the likelihood of recovery.
