# Supplementary material for: Nutritional outcomes of therapeutic feeding program and its predictors among undernourished adult HIV positive patients at healthcare facilities of West Guji Zone, Southern Ethiopia: A retrospective cohort study
Source: PLoS One. 2024 Jan 23;19(1):e0297436. doi: 10.1371/journal.pone.0297436 (PMC10805293; doi:10.1371/journal.pone.0297436)
Supplement: Attachment — Submitted filename: response to reviewers.docx [file pone.0297436.s004.docx]

Title; Nutritional outcomes of Therapeutic feeding program and its predictors among undernourished adult HIV positive patients at healthcare facilities of West Guji Zone, Southern Ethiopia: A Retrospective Cohort Study

Manuscript number: PONE-D-23-28673

Revision Version: 1

Editor’s Decision Received Date: 31/10/2023

Revision Submission Date: 29/12/2023

**Editor comment**

1. Please ensure that your manuscript meets PLOS ONE's style requirements, including those for file naming.

Author response

- We did that our manuscript meets PLOS ONE's style requirements.

2. We suggest you thoroughly copyedit your manuscript for language usage, spelling, and grammar.

Author response

- We carefully proofread our work for grammatical errors.

3. In your Data Availability statement, you have not specified where the minimal data set underlying the results described in your manuscript can be found. PLOS defines a study's minimal data set as the underlying data used to reach the conclusions drawn in the manuscript and any additional data required to replicate the reported study findings in their entirety. All PLOS journals require that the minimal data set be made fully available.

Author response

- We uploaded our study’s minimal underlying data set as Supporting Information files

**Reviewer 1**

**Reviewer Comments:**

1. The paper is well written but the language, grammar and punctuation needs to be improved significantly.

**Author Response:**

We really appreciate your time in reviewing this paper, and we have made an effort to improve the language, grammar, and punctuation.

2. This section is uninformative because it does not review the literature on the main issue of the study. The authors mainly focused on the prevalence of HIV particularly, in the first paragraphs, while the main issue is nutritional recovery among HIV/AIDS patients,
   • Highlight on the epidemiological burden of under nutrition among people living with HIV/ AIDS in terms of its prevalence / rate and the relationship between the two in the background section.

**Author Response:**

We acknowledge this feedback and have attempted to include the study's primary concern, which is the under nutrition burden among HIV/AIDS patients.

3. Results

Figure 1 is not self-explanatory. The X & Y axes are not labeled.

**Author Response:**

We accept the comment and it already corrected and it is self-explanatory. In page 17

- Discussion

There are same vague statements in the discussion section. For example, the statement that says “Age was a further socio-demographic variable connected to nutritional treatment recovery”. This statement needs to be rephrased.

**Author Response:**

We accepted the comment rephrase as;

- Ages of the participants were significantly related with nutritional treatment recovery. In page 26, line 434
- Conclusion

Line 469: The statement that says Nutritional recovery in this study fell short of WHO Sphere requirements.

**Author Response:**

- Thanks for your response and it rephrased.

**Reviewer 2**

1. The manuscript has many editorial problems and needs language editing.

**Author’s response;**

- We sincerely appreciate you taking the time to examine this work, and we have worked hard to fix certain editing issues and improve the language.

Abstract:

1. In the background section of the abstract, the problem under study is not well stated. I suggest rewriting it to show what the problem or gap is and why it needs to be studied.

**Author Response:**

- We tied to highlight the issue or the problem and also incorporate the important of this study in this section.

2. The abstract is lengthy and can be made shorter by minimizing unnecessary details in the methods section.

**Author Response:**

- We acknowledge your feedback and have made an effort per Plos one criterion.

3. The conclusion does not align with the results. For instance, you have concluded that divorced marital status was a negative predictor but you didn’t mention it in the result section.

**Author Response:**

- In line 446, Divorced participants had a 79% lower chance of recovering from malnutrition after enrolling in food by prescription therapy as compared to single participants.

Introduction:

1. Line 58. From the sentence “The human immunodeficiency virus and acquired immune deficiency syndrome……”, each word should be capitalized and put the abbreviation in bracket

**Author Response:**

- We accept and corrected in line 47

2. From Line 67 to 68, the authors mentioned that HIV/AIDS affects 67% of people worldwide. I think this figure is exaggerated and I doubt on the credibility of your source. Check it again.

**Author Response:**

- accounts for 1 in every 25 adult (3.2%) living with HIV and accounting for more than two-thirds of the people living with HIV globally

Reference;

- AB G, AE G, DB K. Malnutrition and Associated Factors among Adult People Living with HIV/AIDS Receiving Antiretroviral Therapy at Organization for Social Service Health Development in Jimma Town Oromia Region South West Ethiopia. Gen Med Open Access. 2018;06(02):4–11.
- African TWHO. Key facts HIV. 2020;

3. Abbreviations should be written in full words in their first use.

**Author Response:**

- We appreciate your feedback on this matter, and it has been fixed.

4. There is a problem of coherence. The authors discuss about the treatments of malnutrition first (line 72 to 97), and then they discuss about the magnitude and effect of malnutrition (line 99 to 107). I suggest discussing the magnitude and effect of malnutrition among HIV patients first.

**Author Response:**

- We made an effort to rectify your perception. We talked about the extent and impact of malnutrition on HIV patients before moving on to how it can be treated.

5. I believe that the problem under study (undernutrition among HIV) is not well stated. Rather the authors focus on the magnitude of HIV and undernutrition treatment procedure.

**Author Response:**

- This also managed

Methods:

1. The study period for a follow-up study should be the time between the initiations of follow up and end of follow up. The authors mentioned the time when they access the patients chart.

**Author Response:**

- We appreciate your comment on this issue and we tried to include this on operational definition as;

**Study period**; the actual data collection (record review from patient cards and registration books) was conducted from February 1-30, 2023.

**Follow-up (period of observation)**: The number of months the HIV patients enrolled in the therapeutic feeding program were followed from the starting of therapy until they developed the event of interest was between January 1, 2018 and December 30, 2022.

In page number 9**.**

2. The study populations of this study were “All under-nourished HIV-infected adults registered in therapeutic feeding centers of public hospitals in West Guji Zone between January 1, 2018 and December 30, 2022” and the data were collected from February 1-30, 2023. But in order to assess whether the participant is recovered or not it needs a minimum of 3 months after initiation of therapeutic feeding based your operational definition. So, there should be at least a 3 months gab between the last date of subject recruitment and outcome assessment. What is your explanation here?

**Author Response:**

- As you mentioned above our study population was all under-nourished HIV infected adults registered in therapeutic feeding centers of public hospitals in West Guji Zone between January 1, 2018 and December 30, 2022. Since this study method is retrospective, the follow up period is from January 2018 to December 2022

3. In the inclusion criteria, why do the authors need to know the nutritional treatment outcomes in order to include the participants in the study? There could be selection bias if we included participants after knowing their outcome status.

**Author Response:**

- The authors not focus on nutritional outcome rather focus on data completeness. It was writing error has been rectified.

4. The authors didn’t calculate the sample size using appropriate formula for survival analysis.

Author response

- Thank you for your kind thoughts and recommendations. We took your comments into consideration. As a result, we used a double population proportion formula for survival analysis and adjust our sample size calculation.

5. I think the data collectors were health care providers working in the ART clinic. This could introduce observer bias, so how do you manage it?

Author response

- It's a fair concern, and we made an effort to reduce bias by using a variety of strategies, such as: providing training and having the lead investigator choose study participants at random; their only responsibility was to gather data.

5. The authors stated that the dependent variable was “nutritional treatment outcome (recovered or unrecovered)”, means it is a binary outcome. So why you conduct survival analysis? If you want conduct a survival analysis, you need to have a time to event data. Please make it clear.

Author response

- I appreciate your kind remarks. We acknowledge your feedback. we had carried out a survival analysis and reported information on the incidence rate of recovery and median duration of recovery for HIV/AIDS patients receiving treatment at West Guji Hospital. Furthermore, we are also disclosing the binary outcome of nutritional treatment (recovered or unrecovered).

7. The authors operationalized adherence. But is it adherence to ART or Adherence to food by prescriptions? I think both are important

**Author Response:**

- To the reviewer: We appreciate your feedback. With regard to your point, we operationalized it as adherence to ART; however, it would be more accurate to characterize malnutrition ( food by prescription adherence) as "defaulter," "non-responder," or "lost to follow-up," which is what we tried to make clear in the operational description.

8. The authors need to define the time to event and censor if they want to stick with survival analysis. They didn’t mentioned when they start and stop following the participants.

**Author’s response**

- We appreciate your feedback and have included it in the operational definition as follows: Follow-up (observation period): The HIV patients who were engaged in the therapeutic feeding program were monitored for the number of months between January 1, 2018, and December 30, 2022, from the beginning of therapy until the development of the event of interest.

Results:

1. The minimum and maximum follow up period, median follow up period, and the total person time contribution of the study participants were not mentioned.

Author response

- Study participants had minimum of 1 month and maximum follow-up period of 10 months with median follow-up of 3 months (IQR 3-5 months), during which time 198 (56.9%) HIV-positive adults recovered from malnutrition after receiving prescription nutrition therapy.

In page number 16

2. Make tables up to the standard by reducing lines

Author response

- I am grateful for your recommendations and good sentiments. We have taken note of your comments. Consequently, we made an effort to standardize the table by eliminating unnecessary tables.

Discussion:

1. Start by summarizing your finding, for instance line 404 you just start with comparison

**Author Response:**

- We appreciate your feedback and have attempted to revise it.

2. The discussion is poorly written which lacks adequate comparison and justifications.

**Author Response:**

- We tried to modify the discussion section and include some rationale for the comparison in response to your input, which we greatly appreciate.

6. From line 464 to 465, the authors mentioned the following as a limitation. “The absence of a control group for comparison and the possibility of data recruitment bias resulted from the samples being restricted to only people with recorded nutritional results.” How could this limitation affect the study? What kind of control group you could use?

**Author Response:**

- We accepted the comment and deleted it despite our best efforts to clarify that the study only included a single retrospective cohort.

Conclusion:

1. From line 469 t0 471, the authors conclude that “The likelihood of nutritional recovery is increased by divorce, WHO clinical stages I and II at admission, working functional status, and age between 18 and 39.” But the result doesn’t support that divorce increases the likelihood of recovery.

**Author’s response**

- Big sorry, this was editorial problem and corrected as;

“Being divorced and having WHO clinical stages III and IV at admission was associated with negative predictors of nutritional recovery while, having working functional status, and being between the ages of 18 and 39 were positive predictors.” From line 503 to 506
